# A Multi-Gage-Length Tensile Specimen for Simultaneous Multi-Strain-Rate Testing

**DOI:** 10.3390/ma19214660

**Published:** 2026-11-01

**Authors:** Javier Geiser, César Garrido, Rodrigo Valle, Víctor Tuninetti

**Affiliations:** 1 Mechanical Engineering Program, Universidad de La Frontera, Temuco 4811230, Chile; j.geiser01@ufromail.cl; 2 Department of Mechanical Engineering, Universidad del Bío-Bío, Concepción 4081112, Chile; cgarrido@ubiobio.cl; 3 Construction Multidisciplinary Research Group, Facultad de Arquitectura, Construcción y Medio Ambiente, Universidad Autónoma de Chile, Talca 3460000, Chile; rodrigo.valle@uautonoma.cl; 4 Department of Mechanical Engineering, Universidad de La Frontera, Temuco 4811230, Chile

**Keywords:** heterogeneous strain fields, digital image correlation (DIC), constitutive parameter identification, transient history effects, intermediate strain rates, virtual extensometry

## Abstract

Characterizing the strain-rate sensitivity of metallic alloys is critical for developing accurate constitutive models, yet traditional sequential methodologies inevitably introduce complex transient history effects. This study proposes a novel Multi-Gage Length (MGL) tensile specimen designed to overcome these limitations by inducing a simultaneous multi-rate kinematic spectrum within a single testing event. Functioning as a multi-ligament array, the geometry is designed to generate different local engineering strain rates across sub-gages of varying lengths within a single loading event. The methodology was validated using SAE 1020 low-carbon steel, with apparent macroscopic responses aligning with established monotonic loading models. Digital image correlation was employed for kinematic tracking; although full-field mapping was constrained by pixel-resolution boundaries in narrow ligaments, a point-to-point virtual extensometer approach successfully captured a nearly six-fold strain rate divergence between central and peripheral sections. Experimental results further identified a critical boundary condition regarding universal testing machine acceleration, revealing that failure in high-speed nominal trials often occurs entirely within the acceleration phase. Supporting rate-independent finite-element analysis revealed heterogeneous deformation and evolving strain-rate ratios, establishing stage-dependent kinematic partitioning among the ligaments. Ultimately, this framework increases experimental information density and provides a potential basis for future inverse finite-element identification of rate-dependent constitutive parameters.

## 1. Introduction

Characterizing the mechanical behavior of engineering materials under dynamic loading conditions is a fundamental requirement for the development and calibration of constitutive models used in advanced numerical simulations [1,2,3,4,5,6,7]. Several engineering materials exhibit marked sensitivity to strain rate, which directly influences their strength, ductility, and failure mechanisms under impacts, explosions, or collisions [8,9,10,11]. In manufacturing processes such as metal forming, stamping, or machining, where local strain rates can span several orders of magnitude, accurate constitutive descriptions that account for strain rate sensitivity are equally critical to ensure reliable numerical predictions [12,13,14,15,16]. Therefore, reliable characterization of strain rate is essential to ensure structural integrity and predictive accuracy in engineering applications [17,18,19,20].

However, conventional methodologies for characterizing strain rate have significant limitations. Generally, discrete experimental setups are required for different strain rates, ranging from universal electromechanical testing machines for quasi-static conditions to split Hopkinson pressure bar (SHPB) systems for high-strain-rate testing [21,22,23,24]. While these approaches are well-established, they require multiple independent experiments [25], which increases cost, time, and material consumption, in addition to introducing uncertainties associated with material variability and inconsistencies between test setups [26]. Furthermore, as highlighted in experimental studies on composite and cellular materials, intermediate strain rate regimes remain particularly difficult to characterize due to the limitations of conventional equipment and the transition between quasi-static and dynamic systems [27,28].

This methodological limitation has driven the development of alternative experimental strategies capable of capturing strain rate-dependent behavior more efficiently [29,30]. Approaches such as stress relaxation and sequential strain rate jump tests attempt to maximize data yield by varying the loading conditions in steps on a single specimen [31,32]. Although these methods allow for understanding strain rate history, these sequential methods introduce complex transient effects and fundamentally fail to achieve simultaneous multi-rate characterization under identical boundary conditions. Similarly, advances in optical metrology have significantly transformed experimental mechanics. Among these techniques, digital image correlation (DIC) has emerged as a powerful non-contact method capable of measuring displacement and strain across the entire field with high spatial resolution [33,34,35,36]. Compared to traditional contact-based methods, DIC offers superior flexibility and enables detailed analysis of heterogeneous strain fields, which is particularly relevant for complex materials and advanced specimen geometries [37]. Furthermore, DIC-based approaches can be integrated with inverse identification methods, such as the Virtual Fields Method (VFM), to extract constitutive parameters from heterogeneous strain fields [38,39].

Despite these advantages, significant challenges remain. Full-field DIC measurements can be significantly affected by spatial resolution limitations, especially in narrow geometries or regions with high strain gradients, leading to noise and inaccuracies [40,41]. Furthermore, under dynamic testing conditions, the reliability of strain measurements depends on the acquisition frequency, synchronization, and camera resolution, which can limit the accuracy of strain rate estimation [42]. These limitations highlight the need for experimental methodologies compatible with optical measurement constraints, while maintaining reliable strain rate characterization. To address these challenges, various studies have explored modified specimen geometries and innovative experimental setups [43,44]. For example, notched or heterogeneous specimens have been used to induce localized strain fields and investigate failure mechanisms under controlled conditions [45]. Experimental studies have also shown that a fracture can occur during the actuator’s acceleration phase, particularly at intermediate strain rates, resulting in invalid data if proper synchronization with the optical measurements is not ensured [46,47]. Similarly, advanced experimental techniques have been developed to extend the capabilities of tensile tests to higher strain rate regimes and improve the accuracy of measurements [48]. In contrast to approaches focused on constitutive identification from heterogeneous deformation fields, the present work investigates the simultaneous measurement of gage-averaged engineering strain rates across predefined measurement lengths within a single slotted tensile specimen using DIC virtual extensometry.

Therefore, a key unresolved challenge in experimental mechanics is the development of specimen designs and testing protocols capable of generating multiple well-defined strain rates within a single experiment. This approach would reduce experimental complexity, improve consistency, and allow for direct comparison of material responses under identical boundary conditions, which remains difficult with conventional multi-test methodologies [23]. Recent studies have shown that the characterization of strain rate still depends on multiple experimental setups and is affected by limitations related to testing equipment, signal accuracy, and boundary conditions, particularly in high- and intermediate-strain-rate regimes [49,50,51,52]. Furthermore, the role of specimen geometry has been identified as a key factor influencing local strain and strain rate distribution, especially in non-standard configurations. Similarly, advances in non-contact measurement techniques, such as DIC, have enabled a detailed analysis of full-field kinematics; however, significant challenges remain regarding measurement reliability, particularly under complex loading conditions, where image quality, spatial resolution, and surface conditions can compromise accuracy [53,54].

To overcome the limitations of sequential testing and spatial resolution constraints, this study proposes and validates a novel non-standard tensile specimen geometry: the MGL specimen. By integrating multiple parallel sub-sections with varying initial calibrated lengths, the MGL geometry subjects distinct microstructural regions to a common loading head displacement, thereby generating a simultaneous spectrum of localized strain rates. This approach is complemented by a point-to-point virtual extensometer methodology based on DIC, which allows for robust and independent kinematic measurements even under dynamic conditions and with limited spatial resolution. The proposed methodology is experimentally validated using low-carbon steel specimens, with special emphasis on the intermediate strain rate regime, where machine acceleration, signal synchronization, and measurement uncertainty can significantly affect data reliability.

Therefore, the novelty of this work lies in two primary contributions. First, the development of a laser-fabricated multi-gauge tensile specimen capable of inducing multiple strain rates in a single test. Second, the implementation of an integrated experimental protocol combining DIC kinematics with traditional mechanical testing to validate these localized deformation regimes. Ultimately, this work establishes the experimental capability of the MGL configuration to generate and measure different engineering strain rates simultaneously under the investigated material, geometric, and loading conditions.

## 2. Materials and Methods

### 2.1. Base Material of the Multi-Gage Length Specimen (MGL)

The material investigated in this study corresponds to a commercial low-carbon steel sheet classified as SAE 1020, with a nominal thickness of 2 mm. This grade is commonly used in structural and manufacturing applications due to its favorable combination of strength, ductility, and formability. The nominal chemical composition of the material is consistent with the specifications provided in SAE J403 [55].

Uniaxial tensile tests were performed in accordance with ASTM E8/E8M [56] using a calibrated *Zwick/Roell* universal testing machine with a maximum load capacity of 100 kN. The tests were conducted under displacement-controlled conditions at a constant crosshead speed of 2 mm/min, corresponding to quasi-static loading conditions. A minimum of three specimens (n=3) were tested under identical conditions to ensure reproducibility. The reported values correspond to the mean value ± standard deviation. Table 1 summarizes the experimentally determined mechanical properties along with the nominal chemical composition of the SAE 1020 steel used in this study. Mechanical properties are reported in SI units, while chemical composition is expressed in weight percentage (wt.%).

### 2.2. Multi-Gage Length Specimen Design and Fabrication

To observe the effects of varying deformation rates within a single testing event, a non-standardized Multi-Gage Length tensile specimen was designed. The geometry takes inspiration from standard plate-type tensile coupons detailed in DIN EN ISO 6892-1 [57] and ASTM E8/E8M [56].

**Figure 1:**
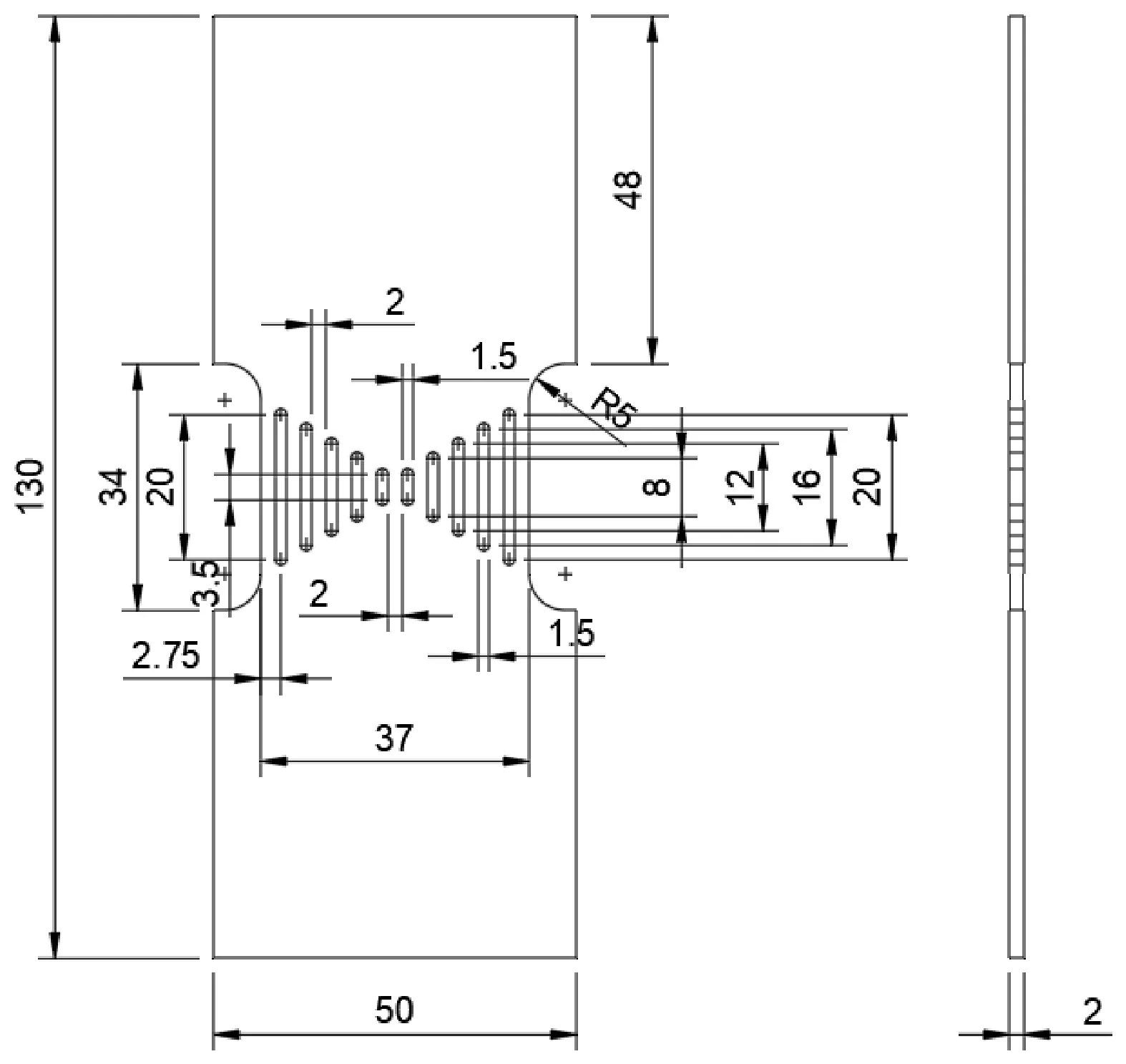
Dimensional schematic of the non-standardized MGL plate-type tensile specimen. The geometry features five discrete sub-sections with variable gage lengths ranging from 3.5 mm (central) to 20.0 mm (peripheral) to induce simultaneous, varying strain rates. All dimensions are provided in millimeters.

The primary innovation of the MGL geometry consists of internal slots that define five parallel sub-specimens (sub-sections) sharing the same total width and thickness but possessing distinct original lengths ($L_{0}$). Moving symmetrically from the periphery to the center of the coupon, the nominal gage lengths are 20.0 mm, 16.0 mm, 12.0 mm, 8.0 mm, and 3.5 mm, respectively. The selected gage lengths span the available specimen gage region and provide different nominal kinematic conditions within a single tensile test. Under the idealized assumption of equal relative opening velocities, the 20.0 mm peripheral and 3.5 mm central gage lengths yield a theoretical strain-rate ratio of 5.71, providing a geometric reference for evaluating the measured kinematic responses. The slot distribution (Figure 1) is symmetrically arranged with respect to the longitudinal axis of the specimen. The gripping regions were designed for compatibility with the jaws of the *Zwick/Roell* universal testing machine.

MGL steel specimens were manufactured via laser cutting using a fiber laser system operating at a cutting power of 1500 W. The process was carried out at a feed rate of 10 mm/min, employing oxygen as the assist gas to enhance the cutting efficiency through an exothermic reaction mechanism. A nozzle with a diameter of 1.0 mm was used to ensure adequate flow and cut quality. These parameters were selected to achieve a stable cutting process, minimizing thermal distortion and ensuring dimensional accuracy of the specimens.

Under the idealized assumption that the relative opening velocity of each sub-gage equals the imposed crosshead velocity (*v*), the corresponding nominal engineering strain rate (${\dot{\epsilon }}_{e}$) is inversely proportional to its initial length ($L_{0}$):

(1) $${\dot{\epsilon }}_{e}=\frac{v}{L_{0}}$$

Following high-precision laser cutting, the internal edges of the slots exhibited significant thermal dross and localized heat-affected zones. To mitigate these potential stress concentrators, the edges of all sub-sections were manually deburred and polished prior to testing.

### 2.3. Mechanical Testing Protocol

Uniaxial tensile tests were conducted using a ZwickRoell Proline Z100 universal testing machine (UTM) equipped with a 100 kN load cell. To characterize the material across a spectrum of kinematic conditions, tests were executed at three nominal crosshead speeds: 10 mm/min, 20 mm/min, and 300 mm/min. These specific velocities were selected to evaluate the specimen’s performance from a quasi-static baseline up to the maximum rated capability of the testing machine. Although standard tensile protocols frequently dictate crosshead speeds of 1–5 mm/min to decouple the material response from universal testing machine compliance, such velocities were not strictly required in this methodology. The integration of high-resolution DIC allows kinematics to be extracted directly from the specimen surface, intrinsically decoupling the measurements from load-train stiffness and crosshead compliance anomalies. A nominal velocity of 10 mm/min (0.1667mm/s) was strategically selected as the quasi-static baseline to balance kinematic validity with optical data synchronization. At this speed, the theoretical local strain rates ($\dot{\epsilon }=v/L_{0}$) across the Multi-Gage array are strictly bounded between a minimum of $0.0083\;s^{-1}$ (within the 20.0 mm peripheral sub-gages) and a maximum of $0.0476\;s^{-1}$ (within the 3.5 mm central sub-gage). These nominal estimates provide a low-speed reference condition for evaluating the MGL kinematic response; however, they do not establish rate-independent material behavior across all ligaments.

For the full-field kinematic analysis, the vertical engineering strain, $\epsilon_{y}$, was derived continuously from the spatial displacement gradient. Specifically, it was computed as the partial derivative of the longitudinal displacement field, *v*, with respect to the initial vertical coordinate, *y*:

(2) $$\epsilon_{y}=\frac{\partial v}{\partial y}$$

Conversely, the point-to-point virtual extensometer methodology intentionally bypassed multi-facet spatial gradients to eliminate boundary decorrelation within the narrowest ligaments. Instead, the localized engineering strain, $\epsilon_{VE}$, was calculated directly from the discrete relative longitudinal displacement of the specific anchored facet pairs:

(3) $$\epsilon_{VE}=\frac{\Delta L}{L_{0}}=\frac{v_{top}\left(t\right)-v_{bottom}\left(t\right)}{L_{0}}$$

where $v_{top}\left(t\right)$ and $v_{bottom}\left(t\right)$ represent the instantaneous vertical displacements of the upper and lower boundary facets of the respective sub-gage, and $L_{0}$ is the initial calibrated reference length between their geometric centroids.

Macroscopic engineering stress ($\sigma_{e}$) and engineering strain ($\epsilon_{e}$) were calculated using the total aggregate initial cross-sectional area of the sub-specimens ($A_{0}=44$ ${\mathrm{mm}}^{2}$) and the instantaneous reaction force (*F*). For visualization, the apparent global engineering response was also transformed using conventional engineering-to-true stress and strain relations:

(4) $$\sigma_{t}=\sigma_{e}\left(1+\epsilon_{e}\right)$$

(5) $$\epsilon_{t}=\ln\left(1+\epsilon_{e}\right)$$

Because the MGL specimen exhibits heterogeneous deformation, these conventional transformations provide apparent global descriptors rather than intrinsic material-level true stress and strain. Their application was restricted to the maximum tensile load, although this restriction does not eliminate the effects of nonuniform deformation.

For the statistical analysis of the strain rate measurements obtained using the DIC technique, the individual MGL specimen was defined as the experimental unit. A total of nine individual specimens were tested: two at 10 mm/min, two at 20 mm/min, and five at a nominal crosshead speed of 300 mm/min. For each specimen, a representative value of the strain rate was determined for each subsection (P1–P5) during the selected analysis interval. Subsequently, the mean and standard deviation (SD) were calculated from the independent specimens within each loading condition. Sequential DIC frames from the same specimen were not considered independent observations due to their temporal correlation. Given the limited number of independent specimens under quasi-static conditions (n=2), no inferential hypothesis tests were performed; therefore, the statistical analysis was limited to descriptive statistics and comparison with theoretical kinematic relationships.

### 2.4. Optical Metrology and DIC Setup

**Figure 2:**
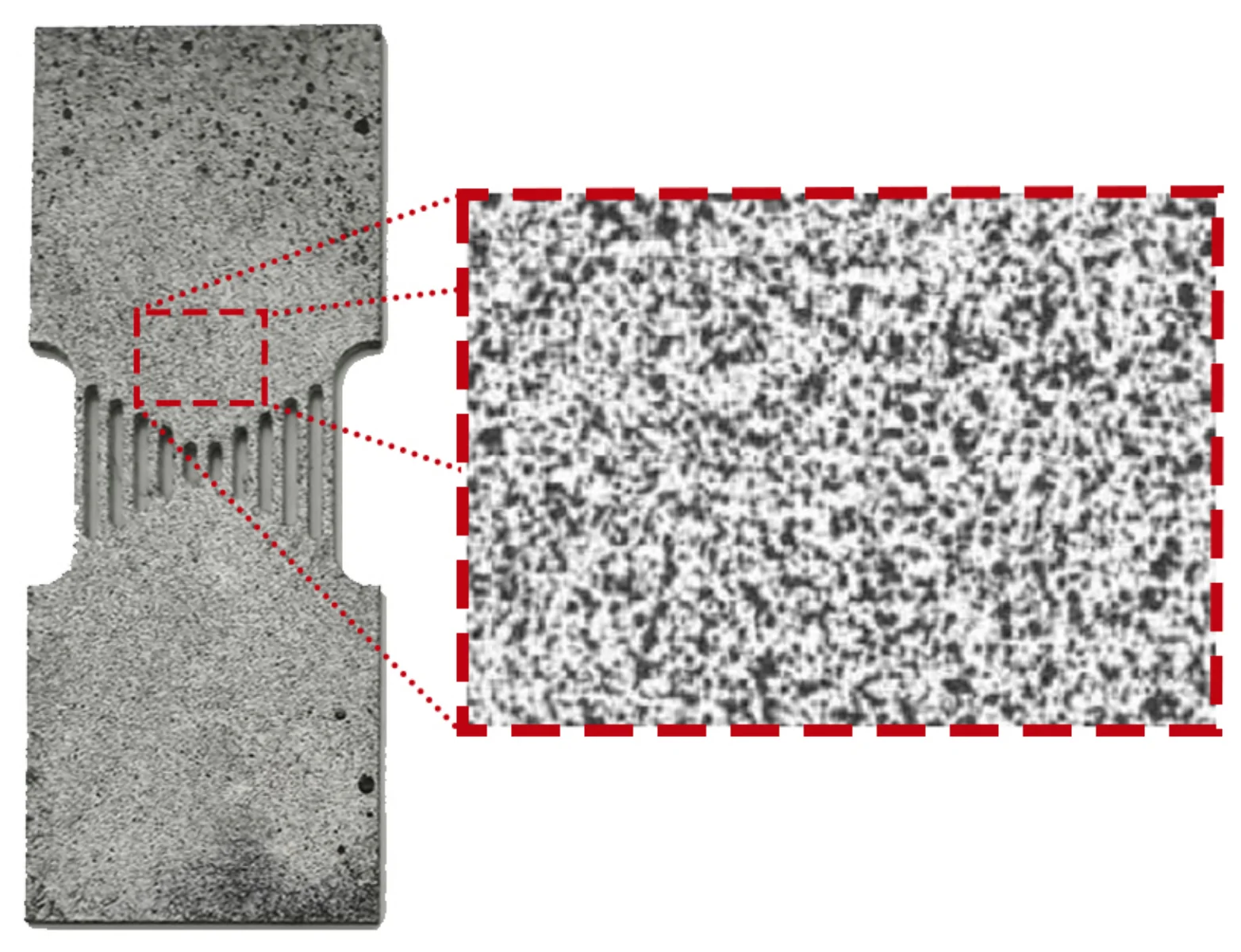
Detail of the optimal 50/50 black-to-white ratio stochastic speckle pattern applied to the specimen surface to facilitate high-accuracy subset tracking during DIC analysis.

Deformation fields and localized kinematic responses were monitored utilizing 2D non-contact DIC. To enable accurate DIC measurements, the specimen surfaces were prepared using a high-contrast stochastic speckle pattern (Figure 2). The application consisted of a two-step aerosol process utilizing matte acrylic paints to minimize specular reflections under active lighting. A uniform white base layer was first deposited, followed by a finely dispersed black mist applied from a controlled distance to achieve an optimal 50/50 black-to-white surface ratio. The resulting speckle density was highly homogeneous across all internal sub-specimens, with an average individual speckle diameter spanning 3 to 5 pixels (approximately 0.4 to 0.7 mm). This specific sizing ensured that each 9-pixel digital evaluation facet contained a minimum of 3 to 4 distinct features, adhering to established best practices for optimal pattern recognition. Consequently, the gradient noise observed during full-field strain mapping was not a product of speckle variation or pattern heterogeneity. Rather, it was a fundamental artifact of the restricted spatial domain; the narrowest 2.0 mm sub-gages corresponded to a width of merely 14 pixels, severely constraining the continuous multi-facet interpolation matrix required for reliable spatial differentiation.

**Figure 3:**
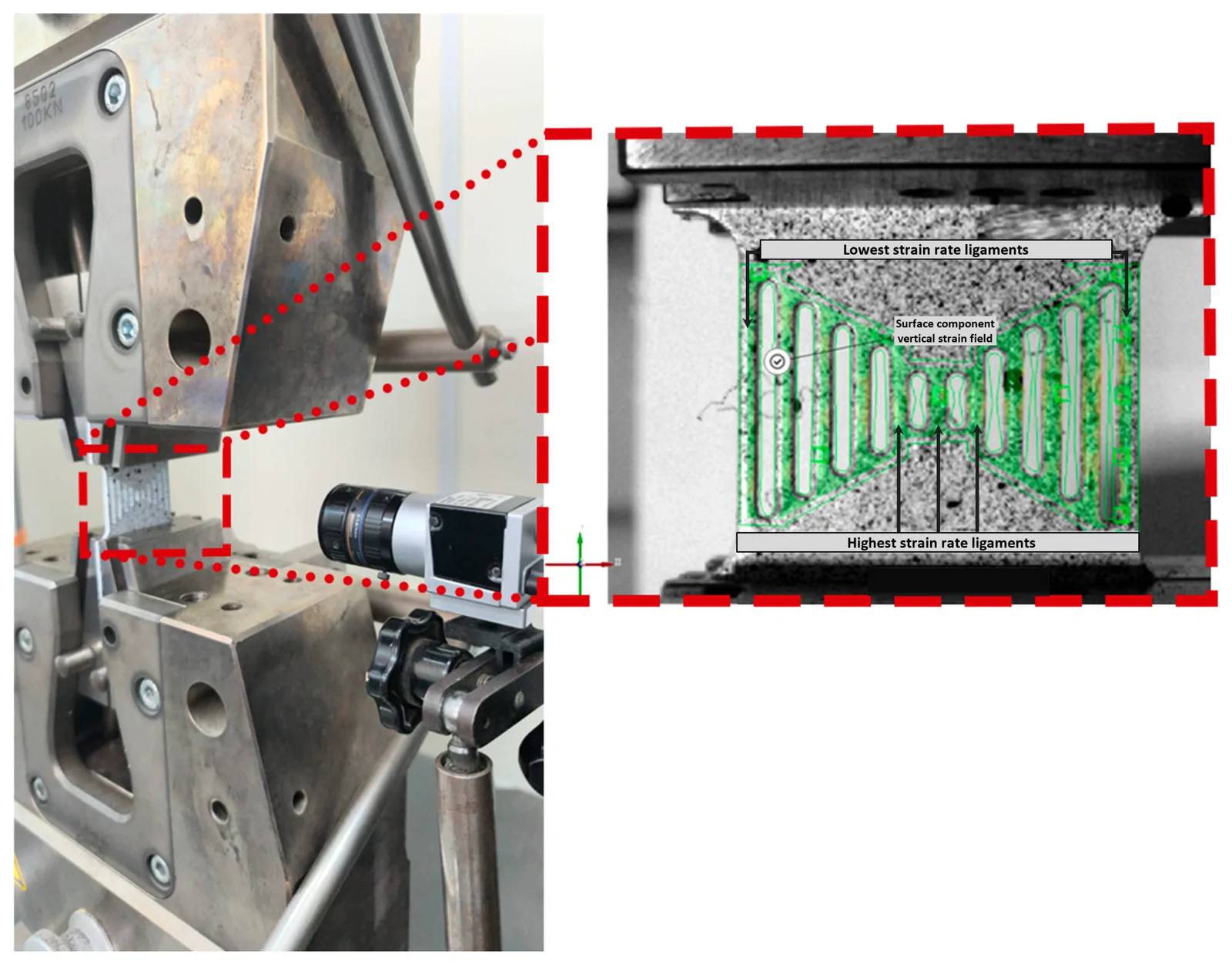
Experimental setup for tensile testing integrated with Digital Image Correlation.

The experimental setup used for the tensile tests is presented in Figure 3, where the DIC system is integrated with the testing machine. The camera was positioned at a fixed distance of approximately 30 cm from the specimen to ensure adequate spatial resolution and field of view. A detailed view of the acquired images is also included, illustrating the digital strain mapping obtained during testing. Optical data acquisition was performed using a Basler acA1300-200uc camera capturing sequential images at an oscillating frequency of approximately 200 frames per second (fps). To maximize the effective pixel density over the target region, the digital field of view was constrained to a 400×400 pixel area focused entirely on the slotted gage section. Image processing and temporal strain computations were executed using Zeiss Inspect Correlate software. The digital surface component was configured with a facet size of 9 pixels and a point distance (step size) of 2 pixels. This optical setup achieved a spatial resolution of approximately 7 pixels/mm, yielding an area of 1.65 ${\mathrm{mm}}^{2}$ per digital facet. Consequently, the narrowest physical width of the structural sub-specimens corresponded to roughly 14 pixels in the imaging domain. To circumvent the severe gradient noise inherent to full-field strain mapping across such narrow digital subsets, localized kinematic divergence was analyzed using a point-to-point virtual extensometer methodology. Digital facet points were anchored at the extremities of each sub-gage to track vertical displacement and compute average sub-section strain.

### 2.5. Finite-Element Modeling and Virtual Extensometry

Finite-element (FE) analysis was conducted in ANSYS Explicit Dynamics to examine the mechanical interactions and time-dependent deformation of the interconnected MGL regions. The model reproduced the specimen geometry with a sheet thickness of 2 mm and a symmetry plane along the specimen length. A constant axial velocity of 0.33333 mm/s (20 mm/min) was prescribed at the upper gripping region, while the lower gripping faces were restrained. The simulated interval was 0–0.50 s, corresponding to an imposed displacement of 0.16667 mm. The numerical analysis represents a forward mechanical investigation of the specimen geometry, rather than an inverse identification of material parameters.

The numerical material response was represented by Johnson–Cook plasticity with the strain-rate coefficient set to C=0 and thermal softening inactive. Under these settings, the flow stress reduces to the rate-independent power-law hardening expression

(6) $$\sigma_{\mathrm{flow}}=\sigma_{0}+K{\left(\varepsilon_{p}\right)}^{n},$$

with $\sigma_{0}=285.9$ MPa, K=499.8 MPa, and n=0.228, adopted from the published steel-sheet parameterization in [10]. With the constitutive strain-rate term disabled, the simulated kinematic partitioning arises from the specified geometry, mechanical interactions, and rate-independent elastoplastic response, without an imposed material strain-rate effect.

The model used four-node tetrahedral elements, with local refinement around the slots and transition regions. The final mesh (nominal element size 0.5 mm) contained 86,656 elements and 19,444 nodes. The mesh-refinement study showed that the maximum equivalent plastic strain decreased from 0.0363 to 0.0322 and 0.0321 for nominal element sizes of 1.0, 0.6, and 0.5 mm, respectively, demonstrating stabilization of the predicted plastic strain response with mesh refinement. A uniform mass-scaling factor of 1000 was employed to improve computational efficiency. The kinetic energy remained at least three orders of magnitude below the internal energy during the evaluated loading interval, while hourglass energy was zero for the employed element formulation.

Six pairs of FE nodes defined axial virtual extensometers P1–P6. Their initial axial separations were $L_{0,i}=$19.590, 15.615, 11.843, 7.774, 3.425, and 3.331 mm, respectively. These are six extracted measurement spans within the specimen, which was designed around five nominal gage-length classes. For each virtual extensometer, the axial displacements of the upper and lower anchor nodes were extracted at 21 equally spaced time points between 0 and 0.50 s, with a sampling interval of 0.025 s. Relative elongation and gage-averaged engineering strain were calculated as

(7) $$\delta_{i}\left(t\right)=u_{i,\mathrm{up}}\left(t\right)-u_{i,\mathrm{lo}}\left(t\right),\;\varepsilon_{i}\left(t\right)=\frac{\delta_{i}\left(t\right)}{L_{0,i}}.$$

Displacement-derived strain-rate histories were evaluated using three-point central differences for interior states and one-sided differences at the interval endpoints. For a time window $\left[t_{a},t_{b}\right]$, the interval-averaged rate and the ratio relative to P1 were calculated directly from the relative displacements:

(8) $${\bar{\dot{\varepsilon }}}_{i,[a,b]}=\frac{\delta_{i}\left(t_{b}\right)-\delta_{i}\left(t_{a}\right)}{L_{0,i}\left(t_{b}-t_{a}\right)},\;{\bar{R}}_{i1,[a,b]}=\frac{{\bar{\dot{\varepsilon }}}_{i,[a,b]}}{{\bar{\dot{\varepsilon }}}_{1,[a,b]}}.$$

The ideal geometric ratio, $R_{i1}^{\mathrm{ideal}}=L_{0,1}/L_{0,i}$, applies only when the corresponding measurement spans have equal relative axial opening velocities. The additional factor $q_{i1}={\bar{R}}_{i1}/R_{i1}^{\mathrm{ideal}}$ quantifies the departure from this condition. The numerical strain-rate histories and tabulated ratios were calculated exclusively from the exported nodal displacements. The differentiated rates are estimates at the stored output resolution (Δt=0.025 s), whereas the interval-averaged rates depend only on the displacement changes between window endpoints.

## 3. Results and Discussion

### 3.1. Apparent Global Mechanical Response

**Figure 4:**
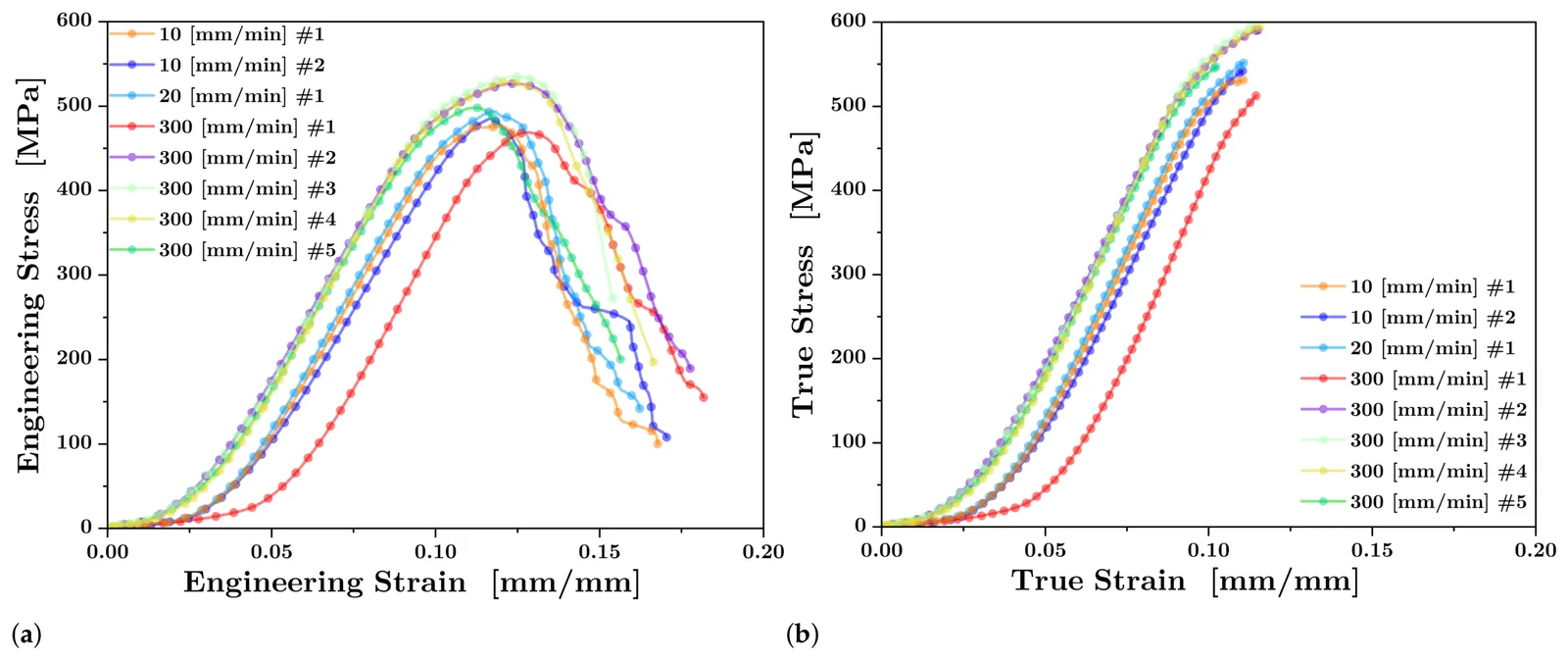
Macroscopic tensile response of the aggregate MGL specimens across varying crosshead speeds (10, 20, and 300 mm/min). (**a**) Engineering stress-strain curves, calculated using the combined initial cross-sectional area. (**b**) Apparent stress–strain curves obtained using conventional engineering-to-true conversions, shown up to the maximum tensile load.

Because the MGL geometry functions as a multi-ligament array rather than a continuous standard coupon, its bulk mechanical behavior is characterized as an apparent global response. This apparent tensile response was evaluated using the aggregate cross-sectional area of the parallel sub-sections (44 ${\mathrm{mm}}^{2}$). The apparent global engineering stress-strain curves demonstrated consistent elastic behavior and yielding across the tested crosshead velocities (Figure 4a). Apparent global yield stresses ranged from approximately 265 MPa during the 10 mm/min baseline trials to 280 MPa at the 300 mm/min nominal speed. Peak reaction forces recorded by the load cell varied between 20 kN and 25 kN. The apparent stress–strain response obtained using conventional engineering-to-true conversions is presented up to the maximum tensile load (Figure 4b). The physical results indicated a highly constrained uniform plastic region; the aggregate specimen exhibited approximately 0.02 mm/mm of global plastic strain between the yield point and the onset of localized necking.

**Figure 5:**
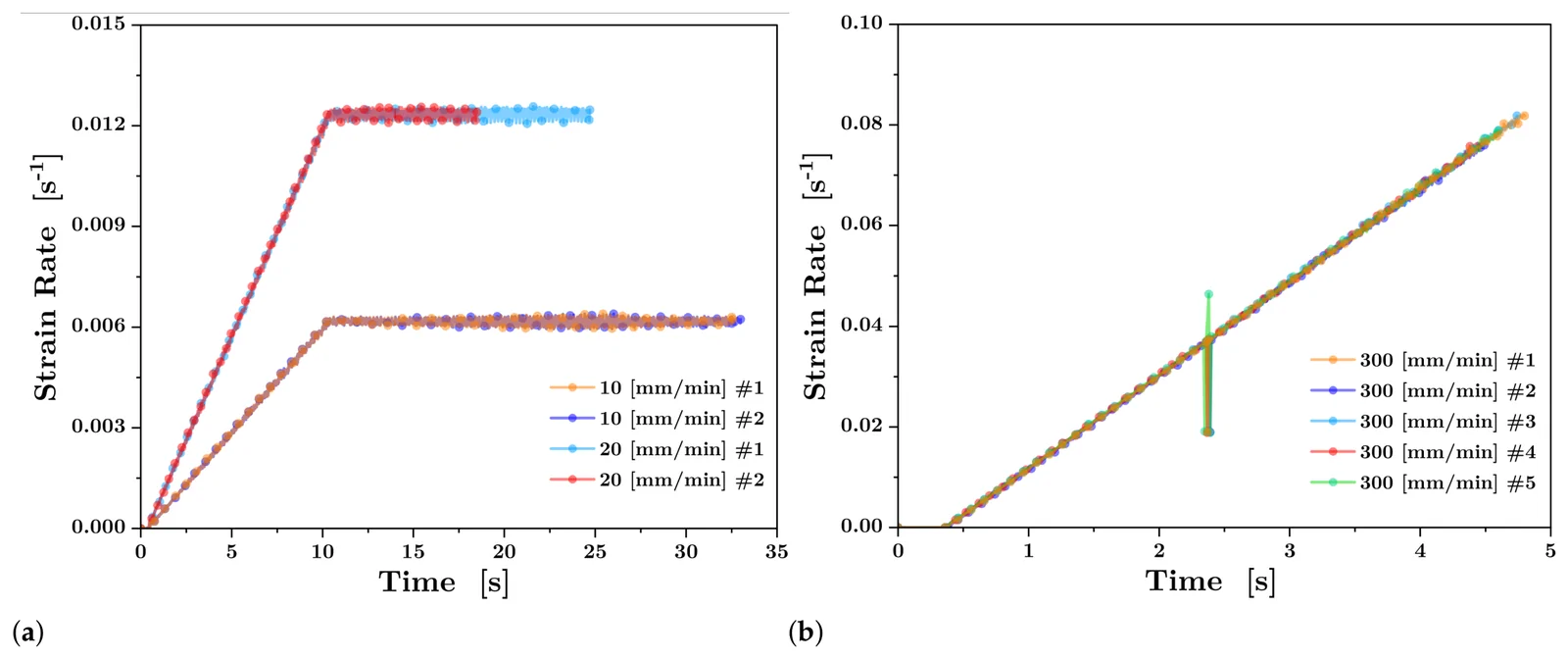
Instantaneous global strain rate profiles derived via backward finite divided differences from the universal testing machine displacement data. (**a**) Low-speed trials (10 and 20 mm/min) achieving steady-state deformation rates. (**b**) High-speed trials (300 mm/min) demonstrating continuous machine acceleration and specimen fracture prior to achieving a constant nominal velocity.

To gain a better understanding of the kinematic behaviour of the experiments, the instantaneous global strain rate was calculated from the machine’s displacement data. The resulting strain rate histories for all tests are shown in Figure 5.

**Figure 6:**
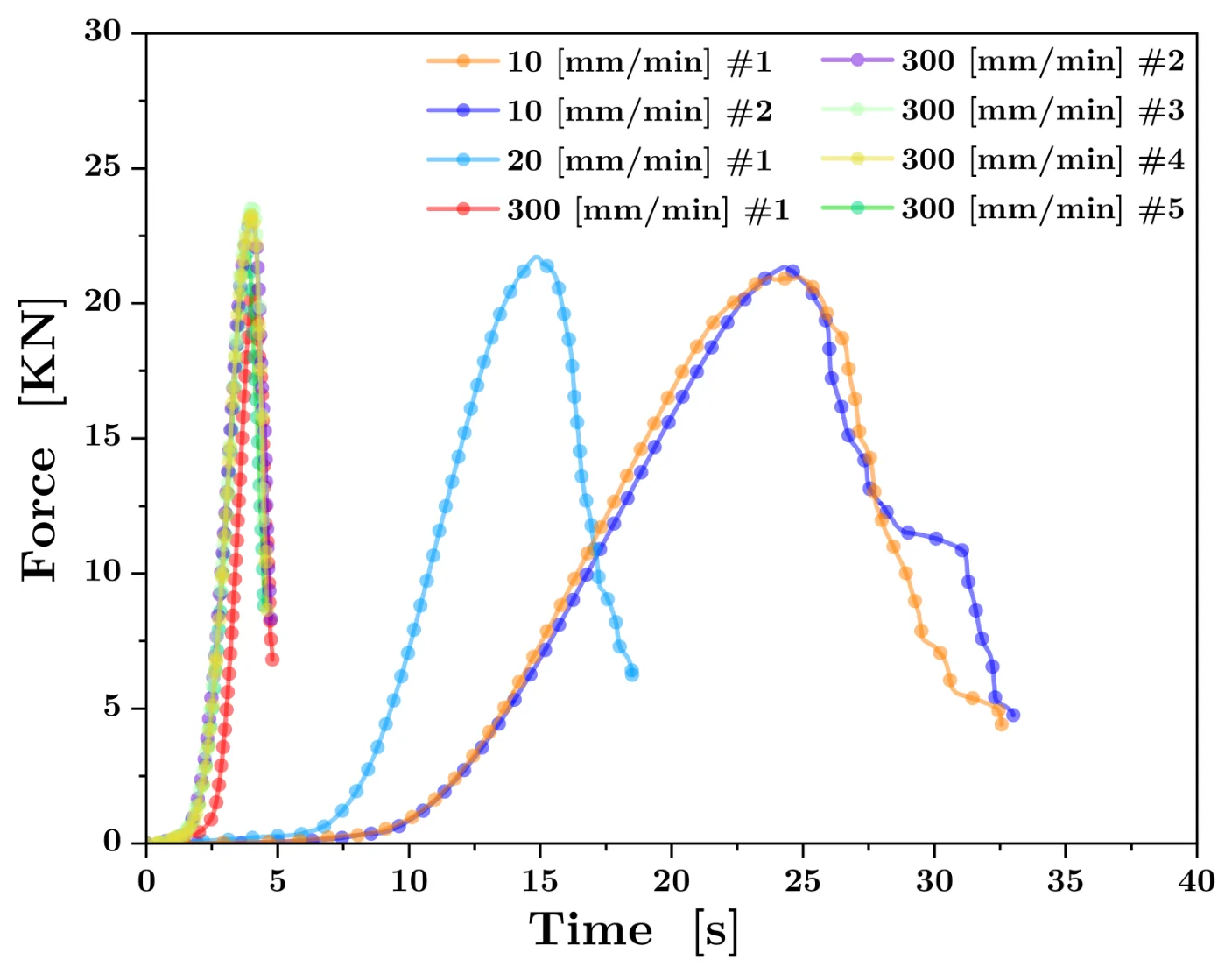
Force-time histories for the MGL specimens across the three tested nominal speeds. The curves highlight the temporal disparity between low-speed tests (>30 s) and high-speed tests (∼5 s to failure).

In low-speed tests (10 and 20 mm/min), the strain rate increases linearly during the initial loading phase and stabilises after approximately 10 s, indicating that the testing machine reaches a constant crosshead speed. In contrast, tests at 300 mm/min do not show a plateau. Instead, the strain rate increases continuously until fracture, demonstrating that the machine remains in the acceleration phase throughout the test. This behaviour becomes more evident when examining the force-time response of the experiments, shown in Figure 6.

The force-time curves highlight the marked differences in duration between the loading conditions. Low-speed tests extend beyond 30 s, allowing for stable deformation. In contrast, high-speed tests reach maximum load in approximately 5 s. This confirms that fracture during tests at 300 mm/min occurs before the machine reaches a constant speed. Consequently, the nominal speed of the test head does not represent the actual strain rate experienced by the specimen.

### 3.2. DIC Kinematic Validation

#### 3.2.1. Full-Field Strain Mapping Resolution Constraints

**Figure 7:**
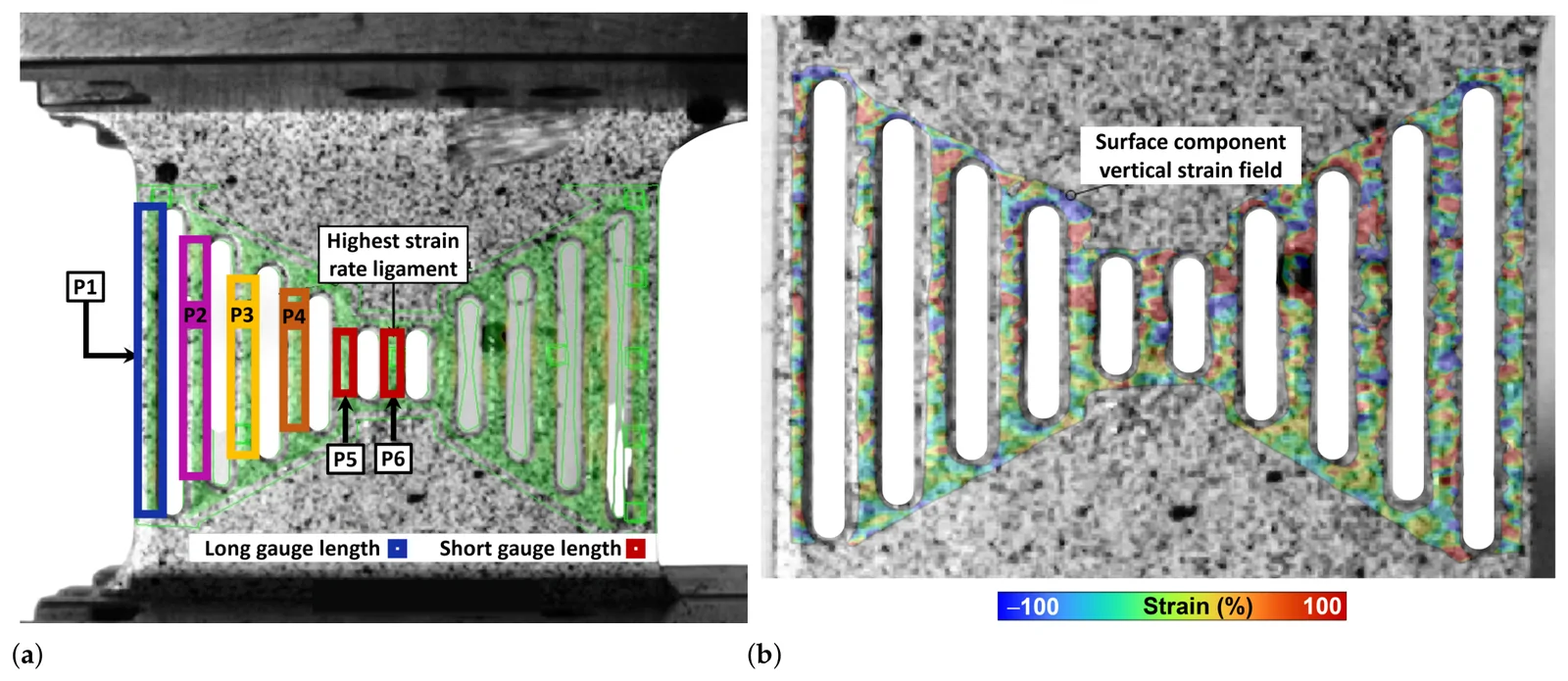
(**a**) Verification of DIC pattern quality using Zeiss Inspect Correlate, showing the mapped digital surface over the MGL specimen and an adequate stochastic speckle distribution, despite the limited pixel width of the sub-gauge regions. The labels P1–P6 identify specific zones subjected to deformation, which are subsequently selected for localized evaluation at different loading velocities. (**b**) Full-field DIC visualization of the vertical strain field, expressed in percent. The restricted spatial resolution within the narrowest ligaments limits the quantitative interpretation of gradient-based strain measurements.

Initial optical evaluations utilized full-field vertical strain ($\varepsilon_{y}$) contours to examine the spatial deformation distribution across the specimen. The digital surface component and the corresponding vertical strain field are presented in Figure 7. The DIC analysis provides a visualization of the heterogeneous deformation across the MGL geometry; however, the quantitative extraction of local strain gradients within the narrower internal sub-gages (Figure 7b) was limited by the spatial resolution of the optical setup.

To maintain the constant 200 fps acquisition frequency required to temporally resolve the simultaneous multi-rate phenomena, the optical field of view was calibrated to encompass the entire macroscopic region, yielding a physical spatial resolution of 7 pixels per millimeter. As a result, the 2.0 mm physical width of the narrowest internal ligaments corresponded to a transverse domain of merely 14 pixels. Given the selected 9-pixel correlation facet, the approximately 14-pixel ligament width provided limited space for independent interior correlation points and constrained the spatial differentiation of the displacement field near ligament boundaries. This severe spatial confinement physically restricts the continuous multi-facet interpolation matrix.

The restricted spatial resolution within the narrowest ligaments also increases the sensitivity of gradient-based strain and strain-rate calculations to optical correlation and numerical differentiation noise. Consequently, the full-field measurements in these regions were not considered sufficiently reliable for quantitative local strain-rate evaluation. A point-to-point virtual extensometer approach was therefore adopted to determine gage-averaged engineering strains from relative displacements, avoiding the need for spatial differentiation across the narrow ligament widths.

For point-to-point virtual extensometry, a 9-pixel facet size and a 2-pixel step size were employed to track the relative displacements of the selected measurement points. These settings were maintained throughout the experimental analysis. The narrow ligament dimensions constrained the available correlation region; however, a systematic facet-size and step-size sensitivity analysis was not performed.

#### 3.2.2. Point-to-Point Virtual Extensometry

**Figure 8:**
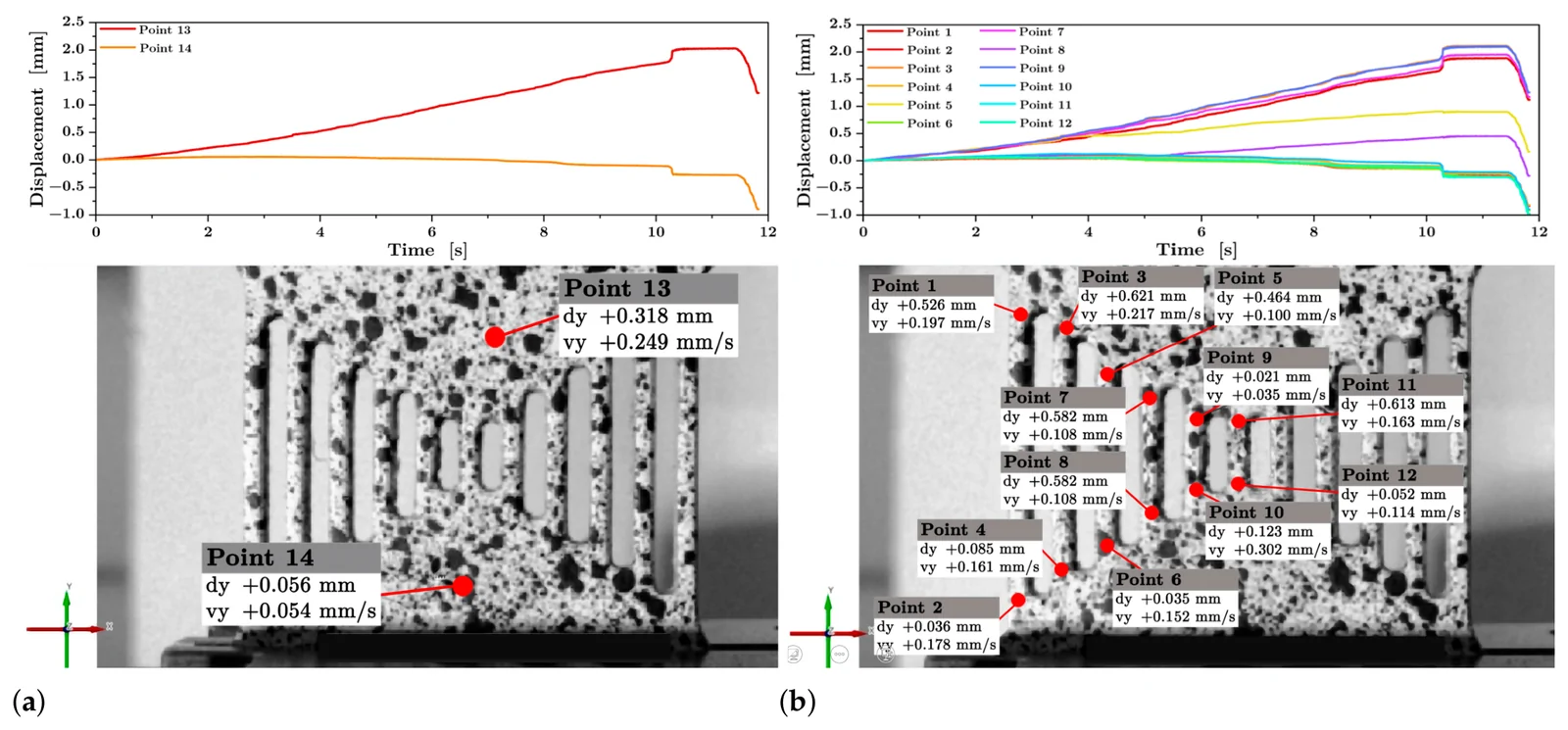
(**a**) Application of the Virtual Extensometer methodology, where paired facet points are anchored at the upper and lower radii of each sub-gage to track vertical displacement and mitigate the noise associated with full-field strain mapping. (**b**) Simultaneous tracking of localized displacement across the MGL geometry, showing the elongation of central (3.5 mm), intermediate, and peripheral (20.0 mm) sub-gages, allowing isolation of the distinct kinematic responses of each section.

To overcome the limitations of full-field mapping, a point-to-point virtual extensometer methodology was implemented. The locations of the selected facets and the procedure for tracking displacement are shown in Figure 8. Digital facet points were anchored at the upper and lower transition radii of each sub-gage, tracking divergent vertical displacements over time.

Pairs of facet points were placed on the transition radii of each sub-calibrator, enabling the vertical displacement of each section to be tracked independently. This method avoids spatial differentiation and instead calculates the average deformation using discrete displacement measurements. Simultaneous tracking of the central, intermediate and peripheral sections allows the kinematic response of each sub-specimen to be isolated. Traditional contact extensometry represents the gold standard for independent kinematic benchmarking; however, the physical attachment of clip-on extensometers or foil strain gauges to the internal 3.5 mm ligaments was strictly prohibited by the experimental setup. Such contact methods would unavoidably obstruct the optical field of view required for simultaneous DIC tracking and introduce severe parasitic stress concentrations along the narrow structural boundaries, artificially triggering premature failure. The measured strain-rate ratios were compared with the ideal geometric prediction of 5.71 for the peripheral (20.0 mm) and central (3.5 mm) gage lengths. This comparison provides a reference for evaluating the kinematic response of the MGL specimen but does not constitute an independent validation of DIC measurement accuracy. Because the ligaments are mechanically interconnected, their relative opening velocities may differ under the same global loading history. Therefore, the actual deformation velocity experienced by any individual sub-gage cannot be assumed equal to the macroscopic machine speed. Instead, an effective localized velocity, $v_{eff}$, must be kinematically reconstructed for each discrete sub-section. This was initiated by isolating the instantaneous relative longitudinal displacement, δ(t), between the upper and lower anchored virtual extensometer facets of the target sub-gage:

(9) $$\delta \left(t\right)=v_{top}\left(t\right)-v_{bottom}\left(t\right)$$

**Figure 9:**
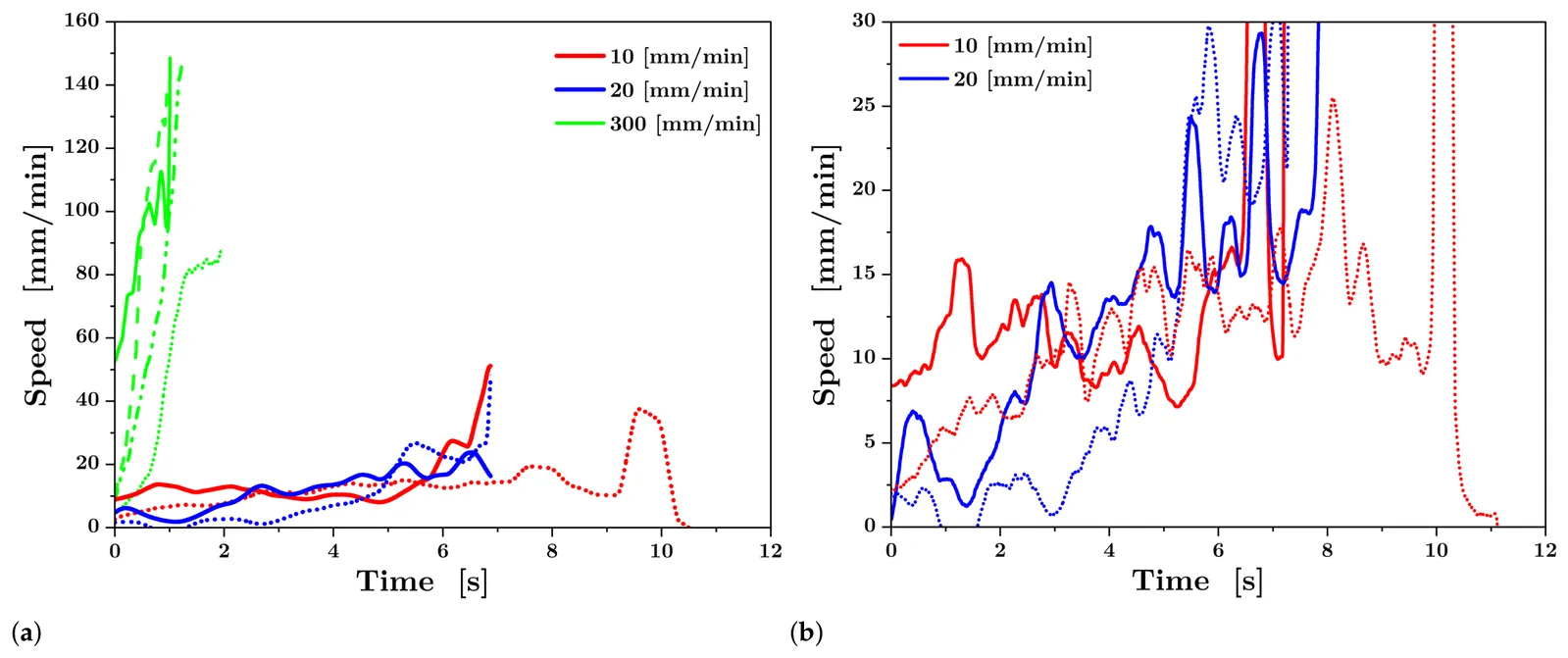
Localized traction velocities determined via point-to-point DIC analysis across (**a**) all tested crosshead speeds and (**b**) low-speed trials specifically. A moving average filter was applied to smooth high-frequency noise inherent to the optical differentiation process.

The effective localized velocity was subsequently derived by evaluating the temporal derivative of this relative displacement. To mitigate the high-frequency noise amplification that inherently occurs when mathematically differentiating the 200 fps optical data stream, the derivative was computed utilizing a standard second-order central difference numerical scheme:

(10) $$v_{eff}\left(t_{i}\right)=\frac{d\delta }{dt}\approx \frac{\delta \left(t_{i+1}\right)-\delta \left(t_{i-1}\right)}{2\Delta t}$$

where $t_{i}$ represents the current discrete temporal frame, and Δt is the constant inverse of the camera acquisition frequency (0.005s). This reconstructed $v_{eff}$ functionally replaces the global machine speed, serving as the true, dynamically accurate kinematic boundary condition for the localized analysis of each sub-gage. The resulting tensile velocity histories are shown in Figure 9.

An assessment of 100 stationary image pairs acquired before loading yielded standard deviations of 2.68 μm in virtual-extensometer elongation and $4.82\times 10^{-2}$ s^−1^ in the instantaneous strain-rate estimates. These values quantify measurement noise under stationary conditions rather than the complete uncertainty of the representative strain rates obtained during loading. In particular, temporal differentiation and smoothing can affect strain-rate estimates, and gage-specific uncertainties during loading were not independently established.

The VE measurements provided experimental evidence supporting the main kinematic hypothesis of the MGL design: the geometry simultaneously induces different local strain rates. According to specimen-level statistics, the average engineering strain rate at 10 mm/min increased from 0.0081 s^−1^ at P1 ($L_{0}=20.0$ mm) to 0.0592 s^−1^ at P5 ($L_{0}=3.5$ mm), demonstrating the marked amplification of the strain rate produced by reducing the length of the measurement subsection.

The velocities obtained using DIC show that the low-speed tests reached relatively stable local strain rates, while the tests conducted at a nominal speed of 300 mm/min exhibited continuous acceleration throughout the loading period. In the latter case, the measured crosshead speed remained below the machine’s nominal setting, as the specimen fractured before the target speed could be fully established. Consequently, the local strain rates experienced by the MGL specimen must be interpreted in relation to the actual kinematic response of the testing system, and not solely based on the nominal crosshead speed. For the quasi-static tests, the strain rate ratios measured between P5 ($L_{0}=3.5$ mm) and P1 ($L_{0}=20.0$ mm) were 7.34 to 10 mm/min and 3.83 to 20 mm/min, respectively. These results demonstrate that the MGL geometry successfully generates substantially different local strain rates within a single specimen. However, the measured relationships do not exactly reproduce the ideal geometric prediction. Thus, the present results provide experimental evidence for the generation of a spatial strain rate gradient; however, determining to what extent the resulting measurements can be used for the independent identification of constitutive parameters requires additional mechanical and inverse finite element method (inverse FEM) analyses.

Under the idealized assumption of equal relative opening velocities across the virtual extensometers, the engineering strain rate is inversely proportional to the initial measurement length. Consequently, the largest theoretical strain rate ratio among the nominal gage length, between the central 3.5 mm ligament (P5) and the peripheral 20.0 mm ligament (P1), is given by:

(11) $${\mathrm{Ratio}}_{\max}=\frac{{\dot{\varepsilon }}_{P5}}{{\dot{\varepsilon }}_{P1}}=\frac{L_{0,P1}}{L_{0,P5}}=\frac{20.0\;\mathrm{mm}}{3.5\;\mathrm{mm}}\approx 5.71$$

To quantify the variability of the measured local strain rates, the analysis was performed at the level of the individual MGL specimen rather than on each individual DIC frame. For each specimen, a representative strain rate value was obtained for each subsection (P1–P5), and the mean and standard deviation (SD) were calculated across the different specimens for each nominal loading condition. A total of nine independent specimens were tested: two at 10 mm/min, two at 20 mm/min, and five at a nominal crosshead speed of 300 mm/min. Consequently, the statistical sample size was n=2, n=2, and n=5, respectively. Individual DIC frames were not considered independent statistical observations due to their temporal correlation. The resulting descriptive statistics at the specimen level are summarized in Table 2.

The standard deviations presented in Table 2 represent the variability among independent specimens for each loading condition, not the temporal variability among individual DIC frames. Given the limited number of independent specimens under quasi-static conditions (n=2), inferential hypothesis tests were not considered sufficiently robust. Consequently, the strain rate results are presented using descriptive statistics and are subsequently compared with the theoretical kinematic relationships derived from MGL geometry. The comparison between experimental and theoretical relationships provides a kinematic assessment of the MGL geometry’s ability to generate spatially differentiated strain rates. The theoretical relationships are determined exclusively from the initial lengths of the measurement subsegments, with values of 1.00, 1.25, 1.67, 2.50, and 5.71 for P1 through P5, respectively. The experimental relationships show the expected increase in local strain rate as the initial length of the subsegment decreases, although deviations from the idealized theoretical values are observed.

To further evaluate the kinematic response of the MGL specimen, the experimental strain rate ratios at points P2 through P5 relative to P1 were calculated and compared with the corresponding theoretical ratios derived from the inverse relationship between the local strain rate and the initial length of the measurement subsection. This comparison allows us to quantify the degree of agreement between the experimentally induced strain rate gradient and the ideal geometric prediction for each subsection and loading condition. The resulting experimental and theoretical ratios, along with their relative differences, are summarized in Table 3.

A comparison of the experimental and theoretical strain-rate ratios in Table 3 confirms that the MGL specimen generates differentiated engineering strain rates within a single loading event. However, the measured ratios deviate from the ideal inverse-length predictions, which assume equal relative opening velocities across the corresponding virtual extensometers. These deviations are consistent with possible load redistribution and deformation interactions among the interconnected ligaments, although the present kinematic measurements do not independently quantify mechanical coupling or isolate its contribution from measurement uncertainty and loading dynamics. Potential geometric strategies for controlling these interactions include adjusting ligament widths and slot spacing, as well as modifying slot-end radii and transition geometries to influence load transfer. The effectiveness of these modifications requires further numerical or experimental assessment.

### 3.3. Kinematic Boundary Conditions in High-Speed Trials

**Figure 10:**
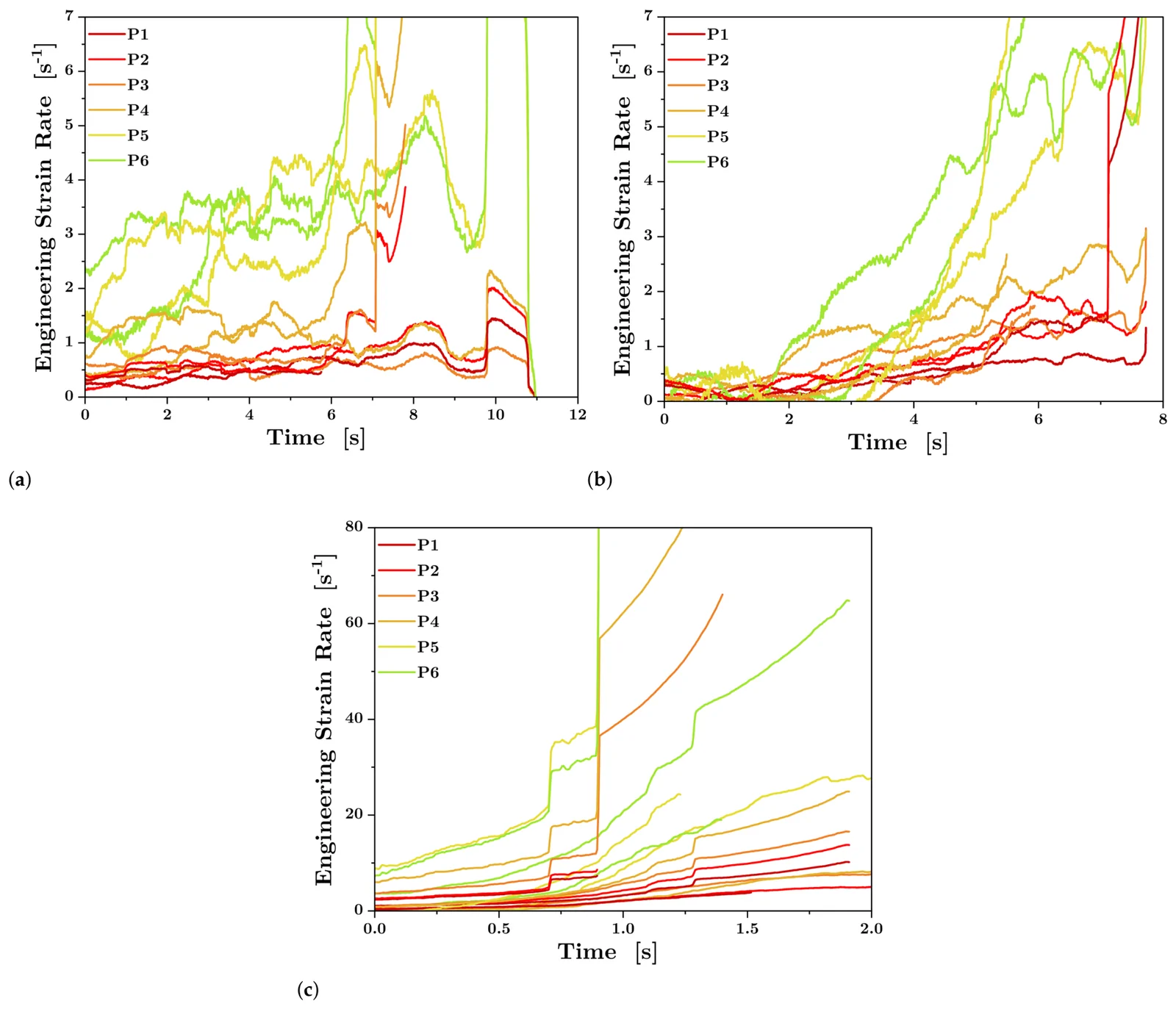
Instantaneous engineering strain rates observed in individual sub-specimens during (**a**) 10 mm/min, (**b**) 20 mm/min, and (**c**) 300 mm/min nominal tests. The measurements demonstrate simultaneous differences in local engineering strain rates, with the observed relationships depending on the loading condition.

Using the validated virtual extensometer methodology, the instantaneous strain rate of each sub-specimen was calculated. The resulting strain rate histories for all sections and loading rates are shown in Figure 10. The results show that reducing the original sub-gage length increases the local strain rate, providing experimental evidence in support of the fundamental kinematic hypothesis of the MGL geometry. For the 10 and 20 mm/min trials, the measured local strain rates exhibited differentiated responses under approximately steady loading conditions, although their ratios did not reproduce the ideal inverse-length predictions.

Although the MGL specimen incorporates five different nominal gage lengths, the present experiments were conducted using a single material and specimen configuration. Consequently, the measured strain-rate distributions are specific to the investigated conditions. Extension to other materials and geometric configurations requires further assessment of their influence on load redistribution and local deformation.

The low-velocity trials at nominal crosshead speeds of 10 and 20 mm/min exhibited approximately steady local strain-rate responses following the initial acceleration period. As reported in Table 2, the measured mean engineering strain rates ranged from 0.0081 to 0.0592 s^−1^ at 10 mm/min and from 0.0128 to 0.0499 s^−1^ at 20 mm/min, with corresponding P5/P1 strain-rate ratios of 7.34 and 3.83, respectively. These results establish the experimentally demonstrated conditions for simultaneous strain-rate differentiation under approximately steady loading in the investigated MGL configuration. In contrast, at a nominal crosshead speed of 300 mm/min, specimen fracture occurred after approximately 5 s, while the testing machine was still accelerating toward its target velocity. Although the measured mean strain rates ranged from 0.0698 to 0.6699 s^−1^, these values correspond to an acceleration-dominated loading regime rather than established steady-rate conditions. Consequently, the operating range of the MGL specimen depends not only on the nominal crosshead speed but also on machine dynamics and the deformation capacity of the specimen.

### 3.4. Finite-Element Analysis of Ligament Interactions and Strain-Rate Evolution

**Figure 11:**
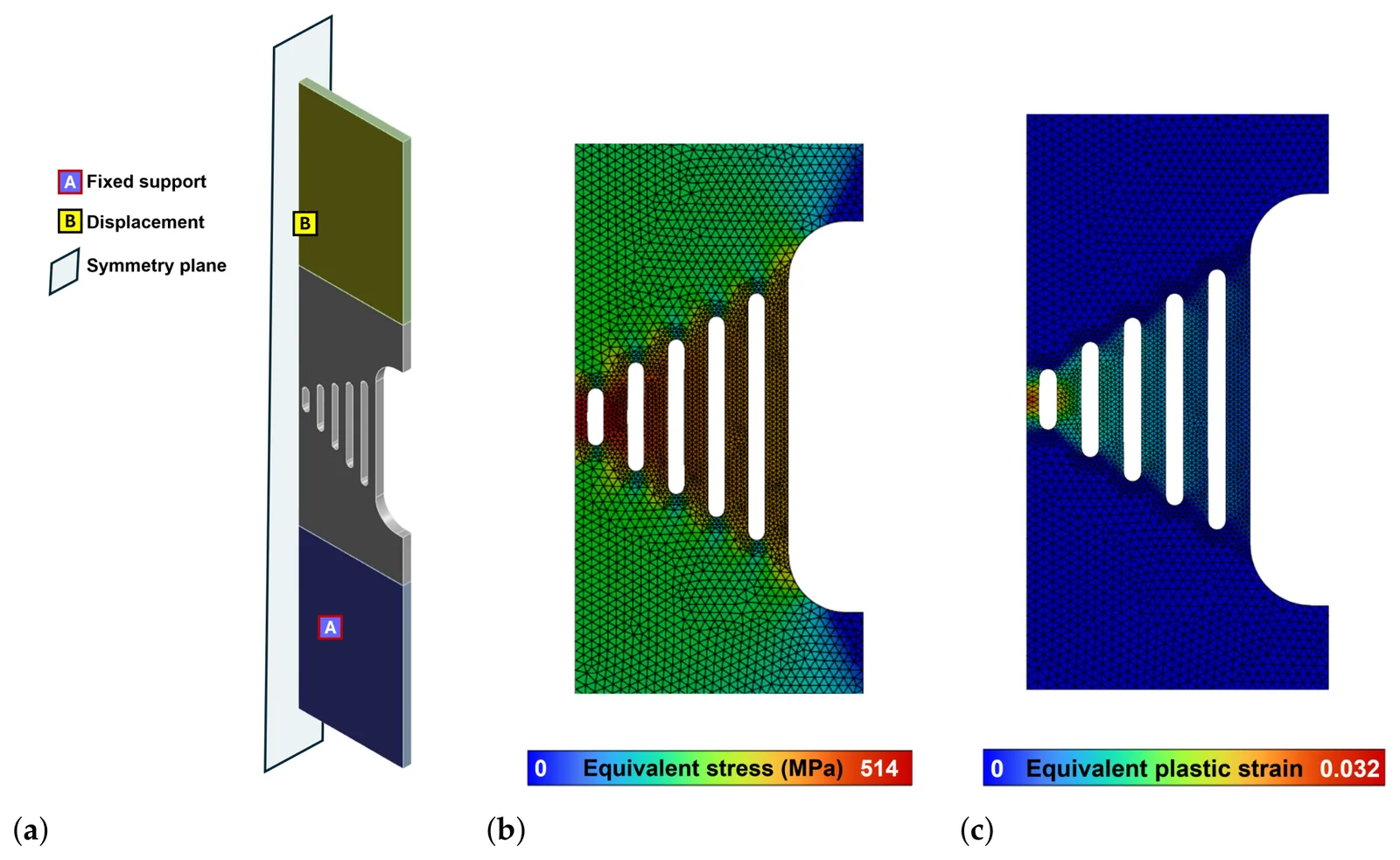
Finite-element model and mechanical response of the MGL specimen: (**a**) three-dimensional model showing the fixed lower grip, prescribed upper-grip motion, and symmetry plane; (**b**) von Mises equivalent stress (MPa); and (**c**) equivalent plastic strain. The fields in (**b**,**c**) correspond to t=0.50 s and an imposed axial displacement of 0.16667 mm.

The numerical stress and equivalent plastic strain fields reveal a heterogeneous mechanical response across the interconnected MGL region (Figure 11). At t=0.50 s and an imposed displacement of 0.16667 mm, the von Mises stress reaches a maximum of approximately 514 MPa, with pronounced spatial variations near the slotted region. The maximum equivalent plastic strain is approximately 0.032. These spatial distributions reveal nonuniform stress transmission and localized plastic deformation within the interconnected geometry, providing a mechanical basis for the differentiated virtual-extensometer responses.

**Figure 12:**
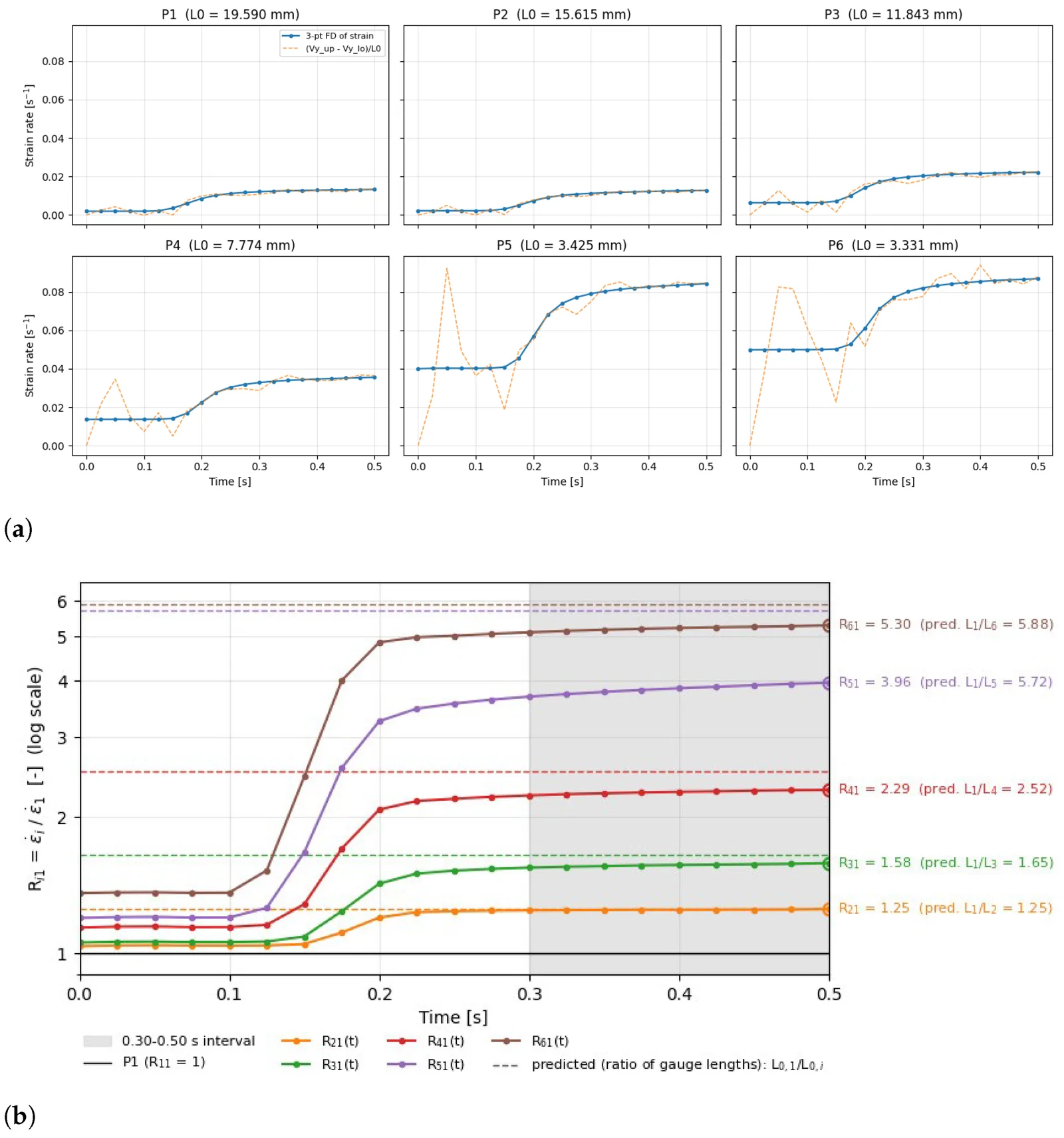
Finite-element virtual-extensometer kinematics over the 0–0.50 s simulation: (**a**) gage-averaged engineering strain-rate histories for P1–P6 calculated by three-point differentiation of relative nodal displacements (solid blue curves) and from exported nodal velocity differences (orange dashed curves); (**b**) instantaneous ratios of the displacement-derived strain rates relative to P1. Horizontal dashed lines in (**b**) indicate analytical inverse-length references based on the assumption of equal relative opening velocities across the virtual extensometers; this assumption was not imposed in the finite-element calculations. The shaded region denotes 0.30–0.50 s. Numerical differentiation was performed without smoothing. The labels at t=0.50 s indicate instantaneous ratios, whereas Table 4 reports ratios of interval-averaged rates.

Figure 12 presents the simulated gage-averaged engineering strain-rate histories derived from the relative nodal displacements of the six virtual extensometers (P1–P6), together with their corresponding ratios relative to P1. Although shorter measurement lengths generally produce higher local strain rates, the ratios evolve substantially during the initial loading stage and cannot be predicted solely from the initial gage lengths. Specifically, the P5/P1 ratio of interval-averaged strain rates increases from 1.22 over 0–0.15 s to 3.84 over 0.30–0.50 s, while P6/P1 increases from 1.42 to 5.22 over the same intervals (Table 4). These values represent ratios of interval-averaged strain rates rather than temporal averages of instantaneous ratios.

During the 0.30–0.50 s interval, the relative-opening factors were $q_{51}=0.67$ and $q_{61}=0.89$, compared with unity under equal average relative opening velocities. The instantaneous strain-rate ratios exhibited smaller variations during this interval, with P5/P1 increasing from approximately 3.69 to 3.96 and P6/P1 from 5.12 to 5.30. This behavior indicates approximately proportional strain-rate evolution during the later loading stage, following the pronounced changes observed earlier in the simulation. The unequal relative opening velocities, together with the heterogeneous stress and plastic strain distributions, demonstrate nonuniform deformation partitioning across the interconnected MGL ligaments. Consequently, the ideal inverse-length relationship provides a geometric reference, while the actual strain-rate evolution reflects the mechanical response of the specimen.

Using a literature-based, rate-independent constitutive model, the FE analysis characterizes the evolution of deformation partitioning among the interconnected MGL ligaments. The experimental DIC measurements demonstrate the simultaneous occurrence of distinct gage-averaged engineering strain rates, while the FE results establish that the corresponding strain-rate ratios evolve during loading rather than remaining fixed by the initial gage lengths. These complementary experimental and numerical findings provide a kinematic foundation for future inverse finite-element identification, in which the coupled displacement histories and constitutive-parameter identifiability can be evaluated.

## 4. Conclusions

The development and testing of the Multi-Gage Length (MGL) tensile specimen demonstrate its capability to generate and measure different engineering strain rates simultaneously within a single loading event, without requiring the imposed rate changes characteristic of sequential testing. Based on the experimental and numerical results, the following conclusions are drawn:

- Functioning as a multi-ligament array, the MGL geometry successfully induces a simultaneous multi-rate kinematic spectrum within a single testing event. The measured engineering strain rates differ among the investigated gage lengths, with the resulting strain-rate ratios depending on the loading condition.
- Full-field strain rate mapping is constrained for sub-specimens narrower than 20 pixels due to gradient-based interpolation noise. However, point-to-point virtual extensometers enable gage-averaged engineering strain and strain-rate histories to be determined from relative axial displacements, providing a displacement-based measurement approach for narrow ligaments where full-field DIC strain mapping is limited by spatial resolution.
- Standard universal testing machines exhibit a critical boundary condition, as they require a significant temporal phase to achieve high nominal crosshead velocities. In the present SAE 1020 MGL experiments at 300 mm/min, specimen fracture occurred during machine acceleration, highlighting the importance of verifying the actual loading conditions when interpreting local strain-rate measurements.
- Supporting rate-independent finite-element analysis revealed heterogeneous stress and plastic-strain distributions and time-dependent strain-rate partitioning among the interconnected MGL ligaments. The P5/P1 and P6/P1 ratios of interval-averaged strain rates increased from 1.22 and 1.42 during 0–0.15 s to 3.84 and 5.22 during 0.30–0.50 s, respectively, demonstrating that the strain-rate ratios are not determined solely by the initial gage lengths.
- The synchronized global force and local DIC displacement histories provide experimental information for future inverse finite-element identification. The applicability of these measurements to constitutive parameter identification requires further numerical and experimental validation.

## Figures and Tables

**Table 1:** Mechanical properties (mean ± standard deviation, n=3) of SAE 1020 carbon steel sheet.

Property	Symbol	Value
Yield strength	$\sigma_{y}$	352±8MPa
Ultimate tensile strength	$\sigma_{UTS}$	468±10MPa
Elastic modulus	*E*	205±5GPa
Elongation	$\varepsilon_{e}$	15±1.2%

**Table 2:** Specimen-level descriptive statistics of the local strain rates obtained by virtual extensometry.

Nominal Speed	Point/Sub-Section	$L_{0}$ (mm)	Mean Strain Rate (s^−1^)	SD (s^−1^)	*n*
10 mm/min	P1	20.0	0.008067	0.000282	2
	P2	16.0	0.012842	0.002663	2
	P3	12.0	0.012991	0.007622	2
	P4	8.0	0.024263	0.012667	2
	P5	3.5	0.059231	0.010333	2
20 mm/min	P1	20.0	0.013010	0.005098	2
	P2	16.0	0.018196	0.006592	2
	P3	12.0	0.012752	0.009353	2
	P4	8.0	0.019099	0.010439	2
	P5	3.5	0.049852	0.006637	2
300 mm/min	P1	20.0	0.069754	0.020771	5
	P2	16.0	0.117492	0.054333	5
	P3	12.0	0.193257	0.150822	5
	P4	8.0	0.241502	0.261496	5
	P5	3.5	0.669924	0.598568	5

**Table 3:** Experimental and theoretical strain-rate ratios relative to P1 for the five MGL sub-sections.

Nominal Speed	Point	$L_{0}$ (mm)	Mean Experimental Strain Rate (s^−1^)	Experimental Ratio vs. P1	Theoretical Ratio vs. P1	Relative Error (%)
10 mm/min	P1	20.0	0.008067	1.000	1.000	–
	P2	16.0	0.012842	1.592	1.250	+27.36
	P3	12.0	0.012991	1.610	1.670	−3.57
	P4	8.0	0.024263	3.008	2.500	+20.31
	P5	3.5	0.059231	7.342	5.710	+28.59
20 mm/min	P1	20.0	0.013010	1.000	1.000	–
	P2	16.0	0.018196	1.399	1.250	+11.89
	P3	12.0	0.012752	0.980	1.670	−41.31
	P4	8.0	0.019099	1.468	2.500	−41.28
	P5	3.5	0.049852	3.832	5.710	−32.89
300 mm/min	P1	20.0	0.069754	1.000	1.000	–
	P2	16.0	0.117492	1.684	1.250	+34.75
	P3	12.0	0.193257	2.771	1.670	+65.90
	P4	8.0	0.241502	3.462	2.500	+38.49
	P5	3.5	0.669924	9.604	5.710	+68.20

**Table 4:** Reference lengths and displacement-derived FE strain-rate ratios relative to P1. Interval ratios are obtained from the end-to-end change in virtual-extensometer elongation over each indicated window. The factor $q_{i1}$ compares the late-interval ratio with the equal-opening-velocity geometric benchmark.

Region	$L_{0,i}$ (mm)	$R_{i1}^{\mathrm{ideal}}$	${\bar{R}}_{i1}$ (0–0.15 s)	${\bar{R}}_{i1}$ (0.30–0.50 s)	$q_{i1}$ (0.30–0.50 s)
P1	19.590	1.00	1.00	1.00	1.00
P2	15.615	1.25	1.04	1.25	0.99
P3	11.843	1.65	1.06	1.57	0.95
P4	7.774	2.52	1.15	2.27	0.90
P5	3.425	5.72	1.22	3.84	0.67
P6	3.331	5.88	1.42	5.22	0.89

## Data Availability

The original contributions presented in this study are included in the article. Further inquiries can be directed to the corresponding author.
